# MXene Enabling the Long-Term Superior Thermo-Oxidative Resistance for Elastomers

**DOI:** 10.3390/polym13040493

**Published:** 2021-02-04

**Authors:** Gui-Xiang Liu, Ya-Dong Yang, Ding Zhu, Yan-Chan Wei, Shuangquan Liao, Mingchao Luo

**Affiliations:** 1 Laboratory of Advanced Materials of Tropical Island Resources, Ministry of Education, Hainan University, Haikou 570228, China; 15543553696@163.com (G.-X.L.); yalkaid@163.com (Y.-D.Y.); 183430@hainanu.edu.cn (Y.-C.W.); 2 School of Materials Science and Engineering, Hainan University, Haikou 570228, China; dingzhu2021@163.com

**Keywords:** elastomers, MXene, thermo-oxidative resistance

## Abstract

The ability of long-term thermo-oxidative resistance is very important for elastomers in application. However, many conventional antioxidants are difficult to realize the long-term thermo-oxidative resistance. To overcome this limitation, a design strategy is introduced by combing elastomers with MXene and natural rubber (NR) is chosen as a model material. MXene is efficient in absorbing oxygen and the generated free radicals in the NR matrix and can inhibit the diffusion of oxygen toward the interior. Moreover, MXene, like graphene and carbon black, absorbs molecular chains, inhibiting the migration of MXene toward the surface of the sample. Such characteristics of MXene endow NR/MXene with the long-term outstanding thermo-oxidative resistance. For example, after three days of the thermo-oxidative process for NR/MXene, the tensile strength is 19 MPa and the retention of tensile strength is 63%, which far exceeds the effects of conventional antioxidants. This work not only provides a good guide for the universal design of elastomers with long-term thermo-oxidative resistance but also expands the application of MXene.

## 1. Introduction

Elastomers, such as natural rubber (NR) and butadiene styrene rubber, are widely used because of their large strain reversible deformability [1,2,3,4]. In application, one of the problems with elastomers is thermo-oxidative aging, leading to the deterioration of properties and early failure. To circumvent this limitation, antioxidants are usually used to prolong the lifetime of elastomers [5]. Although the conventional anti-aging methods improve the ability of thermo-oxidative resistance to some extent, and the mechanism of mixtures antioxidants are also being investigated [6], such conventional antioxidants cannot realize long-term anti-aging effects in the rubber matrix [7]. Further improving the thermo-oxidative resistance and prolonging its lifetime is still a challenge for us.

The loss of antioxidative efficiency is ascribed to antioxidants diffusing toward the surface of elastomer products. To inhibit the migration of antioxidants, previous research often enhances the interactions between antioxidants and matrix, such as the increase of molecular weight for antioxidants [8,9,10], the graft of antioxidants onto polymer chains [11,12], and the immobilization of antioxidants onto the surface of fillers [13,14,15,16,17,18]. However, these methods often require the further complex modification of fillers or polymers, which is limited to the reaction efficiency. For example, the chemistry reaction on the surface of fillers is difficult to be realized and we also have not yet found the direction characterization to analyze the graft efficiency on the surface of fillers. Based on these limitations, it is a challenge for us to further explore another strategy to prolong the anti-aging effect. MXenes, as a kind of two-dimensional early transition metal carbides and carbonitrides, have been widely researched [19,20,21,22,23]. When the size of materials decreases to the nanoscale, such materials often exhibit surprising properties. Under the atmosphere of heat and oxygen, the generated free radicals in the elastomers cause thermo-oxidative aging, resulting in the deterioration of properties. If MXene has the ability to capture the generated free radicals and oxygen, which is similar to the effects of Lignin-carbohydrate complexes [24], MXene may provide an opportunity to improve thermo-oxidative resistance. Moreover, there are often interactions between nanofillers (MXene) and molecular chains, which can inhibit the migration of MXenes toward the surface of samples. To our knowledge, MXenes for thermo-oxidative resistance improvement have not been reported by other groups and there are also no systematic studies on it. 

In this study, our group proposes a strategy to develop elastomers with long-term outstanding thermo-oxidative resistance. NR is chosen as a model material of elastomers. After the introduction of MXene in the NR matrix (NR/MXene), we find that MXene not only absorbs oxygen and the generated free radicals but also absorbs molecular chains. Such characteristics of MXene enable the long-term outstanding thermo-oxidative resistance for elastomer materials. For example, after three days of the thermo-oxidative process for NR/MXene, the tensile strength is 19 MPa and the retention of tensile strength is 63%, which far exceeds the effects of conventional antioxidants. This work not only provides a good guide for the universal design of elastomers with long-term thermo-oxidative resistance but also expands the application of MXene.

## 2. Materials and Methods

### 2.1. Materials

Lithium fluoride (LiF, 99%) and N-tert-butylbenzothiazole-2-sulphenamide (NS, 98%) were provided by Macklin Biochemical Co., Ltd. (Shanghai, China). Titanium Aluminum Carbide powder (Ti_3_AlC_2_, 400 mesh, 98%) was purchased from 11 Technology Co., Ltd. (Jilin, China). The fresh NR latex from clone RRIM600 was provided by Hainan Rubber Industry Group Co., Ltd. (Hainan, China). Zinc oxide (ZnO), sulfur, stearic acid, ethanol absolute (99.5%), formic acid, antioxidants, and hydrochloric acid (37%) were industrial grade.

### 2.2. Preparation of MXene (Ti_3_C_2_T_x_) Nanosheets

**Scheme 1:**
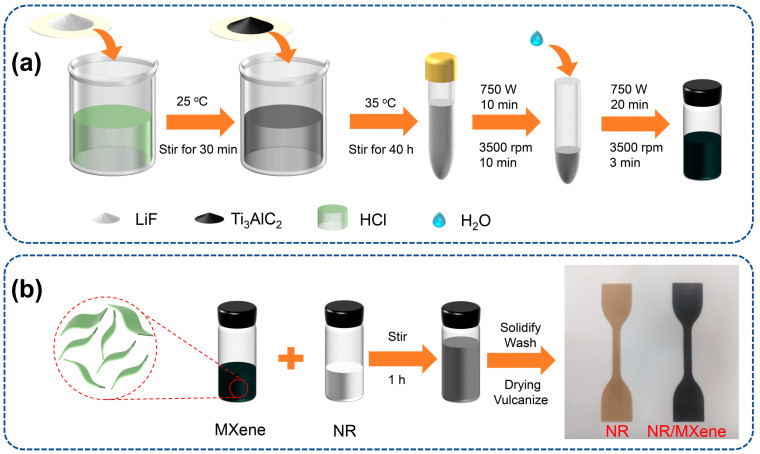
Schematic diagram of the preparation approach. (**a**) MXene (Ti_3_C_2_T_x_) nanosheets. (**b**) natural rubber (NR)/MXene nanocomposites.

Ti_3_C_2_T_x_ was synthesized by selectively etching an Al layer of Ti_3_AlC_2_ with a modified LiF/HCl method (Scheme 1a). Typically, 2 g of LiF was dissolved into 40 mL of 9 M HCl by stirring for 30 min and then 2 g of Ti_3_AlC_2_ was added slowly (30 min) to the above solution (severe reaction, please pay attention to safety). The mixture was further stirred continuously for 40 h at 35 °C. After full etching, the reaction suspension was washed with deionized water and centrifuged for several cycles under 3500 rpm until reaching a pH value of 5. To obtain monolayer MXene, the appropriate amount of anhydrous ethanol was added to multilayer Ti_3_C_2_T_x_ (sediment), which was sonicated (750 W) for 1 h. The monolayer MXene was in suspension. Finally, the colloidal suspension containing Ti_3_C_2_T_x_ nanosheets was obtained and kept at low temperature (4 °C). To increase the yield rate, the lower sediment was sonicated and centrifugated repeatedly.

### 2.3. Preparation of NR/MXene Nanocomposites

The MXene suspension (3 mg/mL) was gradually added into NR latex under continuous stirring for 1 h (Scheme 1b). The compound latex was solidified by formic acid. The coagulated sample was washed in flowing deionized water until reaching a pH value of 7 and the samples were dried to a constant weight under 50 °C for two days. The NR composites were formulated and prepared as follows: rubber (solid content), 100 by weight; Ti_3_C_2_T_x_, 7.0 parts by weight per hundred-part rubber (phr); stearic acid, 2 phr; zinc oxide, 5 phr; sulfur, 2.2 phr; accelerator NS, 1.5 phr. According to the optimum vulcanization time (*t_90_*), the compounds were subjected to compression under 15 MPa at 145 °C. The specimen was a dumbbell-shaped thin strip with central dimensions of 20 mm × 4 mm × 1 mm and then the specimens were suspended in an oven with an air convection environment for different aging times at 100 °C for one, two, three, and five days.

### 2.4. Characterization

The crystalline structure was investigated by X-ray diffractometer (XRD, Rigaku Smart Lab, Tokyo, Japan) with Cu Kα radiation (λ = 0.1540 nm) from 5° to 70°. TEM images for samples were obtained on FEI Talos F200C TEM (Thermo Fisher Scientific, Waltham, MA, USA) at an accelerating voltage of 200 kV. Ultrathin sections for TEM were prepared by an ultramicrotome (Leica EM FC7, Wetzlar, Germany) with a diamond knife at −80 °C. 

X-ray photoelectron spectroscopy (XPS) tests were performed using a K-Alpha XPS (Thermo Fisher Scientific, Waltham, MA, USA) to investigate the changes of O/C atomic ratio in the interior of the sample upon thermo-oxidative process (100 °C for three days) and the changes in content ratios of Ti in different orbital valence states of MXene film upon thermo-oxidative process (100 °C for 1 day). 

Electron paramagnetic resonance spectroscopy (EPR, Bruker A300 spectrometer, Billerica, MA, USA) was used to detect the concentration of free radicals of samples after the thermo-oxidative process (100 °C for three days). The Bruker A300 spectrometer was equipped with an ER4122SHQ microwave cavity and operated at 9.85 GHz and a modulation frequency of 100 kHz.

NR/MXene nanocomposites and neat NR were subjected to Attenuated Total Reflectance Fourier transform infrared (FTIR) spectroscopy (ATR Spectrum Two, PerkinElmer, Waltham, MA, USA) to characterized the change of groups before and after aging. The wavenumber range was from 4000 to 600 cm^−1^. A resolution of 4.0 cm^−1^ was used for all spectral analyses with an accumulation of 16 scans.

The crosslinking density of samples was measured using the equilibrium swelling method. Regular samples of 0.2 g were immersed in toluene at 25 °C. After one week (equilibrium swelling time), network chain density was calculated by the Flory–Rehner equation, as follows:

(1) $$-\ln(1-\phi_{r})-\phi_{r}-\chi_{r}{\phi_{r}}^{2}=nV_{0}({\phi_{r}}^{1/3}-\frac{1}{2}\phi_{r})$$

(2) $$M_{c}=\frac{\rho }{n}$$

where *φ_r_* is the polymer volume fraction in the swollen network, *V*_0_ is the molar volume of the solvent (106.2 mL/mol for toluene), *χ_r_* is the Flory–Huggins polymer-solvent interaction term (0.393 for NR/toluene), *n* is the average number of movable chain segments per unit volume (mol/mL). *M_c_* is the average mass of network chains and *ρ* is the density of NR (0.913 g/mL for NR).

The stress-strain curves of samples were measured with a strain rate of 500 mm/min using a commercial tensile tester (GOTECH AI-3000, Qingdao, China) at room temperature. The needed dimension of samples was a dumbbell-shaped thin strip with central dimensions of 20 mm × 4 mm × 1 mm.

Dynamic mechanical properties were measured in a tensile mode on a dynamic mechanical analyzer (DMA 850, TA Instruments, New Castle, DE, USA) under a nitrogen atmosphere. The DMA measurement was performed with a heating rate of 3 °C/min and a frequency of 1 Hz. The temperature range was from −100 °C to 100 °C. In all cases, a preload force of 0.01 N was applied.

To test the long-term stabilization of MXene for elastomers, the NR/MXene samples were cut into a dumbbell thin strip with a center size of 20 mm × 4 mm × 1 mm which was approximately 1 g. The samples were extracted by 50 °C hot water for three days. Subsequently, the sample was placed in a 100 °C thermo-oxidative environment for one day. As a control, NR/DBH was subjected to the same experimental treatment.

Unvulcanized NR/MXene weighing 0.5 g was cut into small pieces of about 1 mm^3^. The unvulcanized NR/MXene was placed in a stainless steel mesh sieve, which was soaked in toluene at room temperature. The solvent was replaced by fresh toluene every 24 h. To quantitatively characterize the interaction between MXene and the polymer chain, the rubber bond after 72 h was dried in a vacuum drying oven to a constant weight.

## 3. Results

### 3.1. Design Strategy

**Figure 1:**
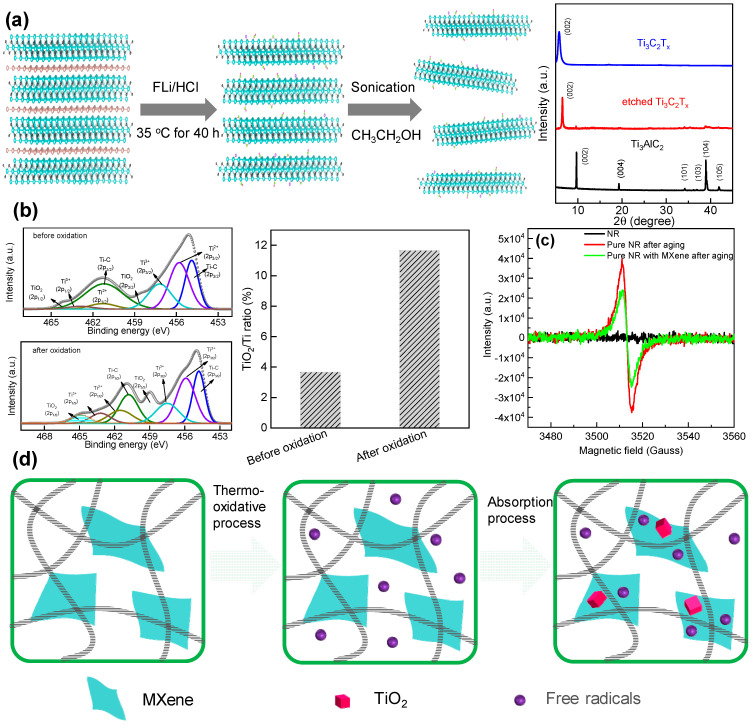
Design strategy of elastomers with outstanding thermo-oxidative resistance. (**a**) Schematic illustration of etching preparation for Ti_3_C_2_T_x_ MXenes. X-ray diffractometer (XRD) patterns exhibit the results of Ti_3_AlC_2_, the etched Ti_3_C_2_T_x_, and the exfoliated Ti_3_C_2_T_x_. (**b**) The oxidation reaction in MXene. (**c**) The first derivative of electron paramagnetic resonance (EPR) spectra for NR, pure NR after aging at 100 °C, and pure NR with MXene after aging 100 °C. (**d**) Schematic illustration of the ability to absorb oxygen and free radicals in the elastomer matrix.

According to previous work, we used LiF/HCl to etch Ti_3_AlC_2_ for obtaining multilayer MXene and then exfoliate multilayer MXene by sonication (Figure 1a) [25,26,27]. The removal of Al layers leads to a shift of (002) peak from 9.6° to 6.5° and further to 5.8° for the exfoliated MXene sheets. After MXene sheets were exposed to a thermo-oxidative environment, some MXene structures transform into TiO_2_. We used X-ray photoelectron spectroscopy (XPS) to obtain the XPS patterns of MXenes before and after oxidation (Figure S1 from Supplementary Material). Further, we abstracted the data of 2p orbitals in Figure S1 (Figure 1b). Such XPS results show that TiO_2_ content clearly increases after oxidation, which confirms that MXene has the ability to absorb oxygen. To prove the ability of MXene to absorb free radicals, pure NR was blended with MXene, which was followed by the thermo-oxidative aging process at 100 °C. NR upon aging exhibits the production of free radicals, while the concentration of free radicals rapidly decreases in the presence of MXene (Figure 1c). Such a decrease in free radical concentration indicates that MXene has the ability to eliminate free radicals. Oxygen and free radicals are the main causes of elastomer aging. Inspired by the feature of MXene absorbing oxygen and free radicals, our group develops elastomers with outstanding thermo-oxidative resistance in this work, as illustrated in Figure 1d.

### 3.2. Changes in Structure during Thermo-Oxidative Process

**Figure 2:**
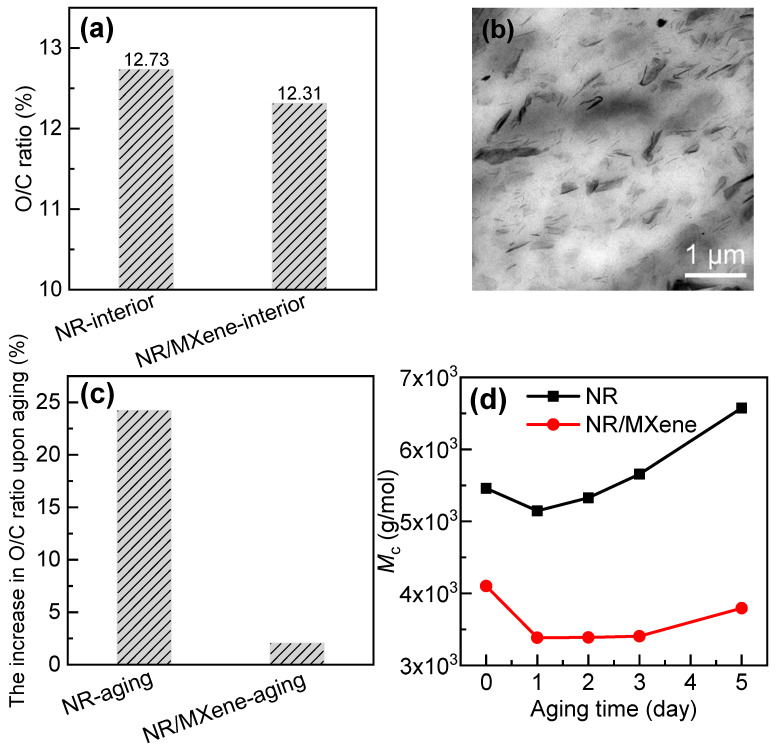
Changes in the structure of NR and NR/MXene composite upon the thermo-oxidative process. (**a**) The O/C ratio of NR and NR/MXene composite in the interior of the sample. (**b**) Transmission electron microscope (TEM) image of NR/MXene composite which shows the dispersion of MXene nanosheets in the NR matrix. (**c**) The increase in O/C ratio of NR and NR/MXene composite upon the thermo-oxidative process. (**d**) The changes in average mass of network chains (*M*_c_) of NR and NR/MXene composite upon different aging times.

Thermal oxidation usually increases the oxygen/carbon (O/C) atomic ratio of the samples. XPS is used to obtain the XPS patterns of samples (Figure S2) and we further investigated the effect of MXene on the O/C ratio for the interior of NR and NR/MXene (Figure 2a). In the sample interior, NR/MXene has a lower O/C ratio than NR. MXene, as a two-dimensional material, is dispersed in the NR matrix (Figure 2b), which hinders the diffusion of oxygen. After the thermo-oxidative process, the XPS patterns of NR and NR/MXene were obtained in Figure S2b and then we abstracted the O/C ratio (Figure S3). To study the degree of oxidation induced by the thermo-oxidative process, we calculated the increased O/C ratio upon aging (Figure 2c). Compared with NR/MXene, NR shows a significantly increased O/C ratio after the thermo-oxidative process. The free radical scavenging, oxygen absorption, and oxygen barrier effects of MXene in the NR matrix contribute to suppressing the oxidation degree of NR/MXene during the thermo-oxidative process. Moreover, the change in molecular chain structure was also analyzed. During the thermo-oxidative process, the scission of main molecular chains and destruction of crosslinks are due to aging [28]. FTIR was used to characterize the changes of groups before and after the thermo-oxidative process (Figure S4a,d). We can notice that NR shows a clear tendency to increase the intensity of the hydroxyl peak as the aging progresses (Figure S4b). In contrast to NR, the intensity of the hydroxyl peak for NR/MXene does not show regular changes (Figure S4e). As the aging time proceeds, MXene can continuously absorb free radicals. The carbonyl peaks (from 1750 to 1600 cm^−1^) of NR/MXene remain almost unchanged with increasing aging time (Figure S4f), while NR shows a clear enhancement of the carbonyl peaks upon aging (Figure S4c). In Figure 2d, the crosslinking structure of NR changes dramatically the thermo-oxidative process, while that of NR/MXene does not exhibit obvious change upon the thermo-oxidative process. Such phenomena are ascribed to MXene being efficient in protecting the sample from thermal oxidation. During the aging process, MXene hinders the diffusion of oxygen and absorbs the free radicals in the NR matrix, protecting the molecular chain. Therefore, the crosslinking density (crosslinking structure) does not exhibit obvious change after different aging times.

### 3.3. Changes in Mechanical Properties Upon Thermo-Oxidative Process

**Figure 3:**
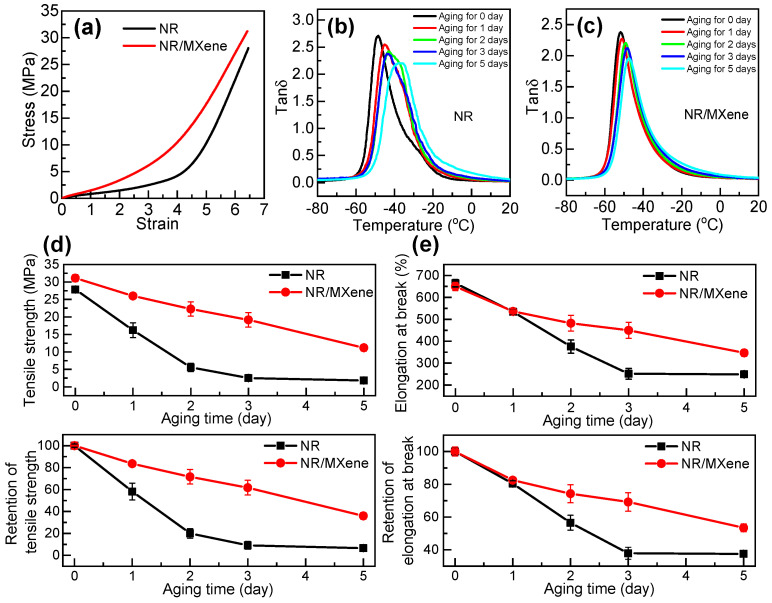
Changes in the mechanical properties of NR and NR/MXene composite upon different thermo-oxidative times. (**a**) Stress-strain curves of NR and NR/MXene composite. (**b**) Temperature-dependent curves of NR upon different aging times. (**c**) Temperature-dependent curves of NR/MXene composite upon different aging times. (**d**) Changes in tensile strength upon different aging times, which contain tensile strength and retention of tensile strength. (**e**) Changes in elongation at break upon different aging times, which contain elongation at break and retention of elongation at break.

Based on the above observations, free radical absorption, oxygen absorption, and oxygen barrier of MXene in the NR matrix are responsible for thermo-oxidative resistance from the microstructure. In this part, we investigated the effect of MXene on thermo-oxidative resistance from macro-performance. After the introduction of MXene into the NR matrix, the matrix shows the enhancement behavior of MXene (Figure 3a), which is similar to the effects of graphene [29,30,31,32]. After molecular chains are attacked by oxygen and free radicals, the topology structure of NR changes, resulting in the changes of glass transition temperature (*T_g_*) upon the thermo-oxidative process (Figure 3b). However, the obvious changes of *T_g_* in NR/MXene does not appear in Figure 3c, due to MXene protecting molecular chains from being attacked by oxygen and free radicals. With the increase of aging time, tensile strength and elongation at break of samples show a decreasing trend (Figure 3d,e). Significantly, NR upon aging process exhibits a very remarkable decreasing trend, compared with NR/MXene. When aging time is three days, the retention of tensile strength and retention of elongation at the break for NR decrease to 10% (2.7 MPa) and 37% (250%), respectively. To our surprise, after three days of aging, the retention of tensile strength and retention of elongation at the break for NR/MXene remain 63% (19 MPa) and 70% (454%), respectively. These results indicate that MXene can act as a promising antioxidant and exhibits outstanding long-term thermo-oxidative resistance.

### 3.4. Long-Term Stabilization of MXene in the Matrix

**Figure 4:**
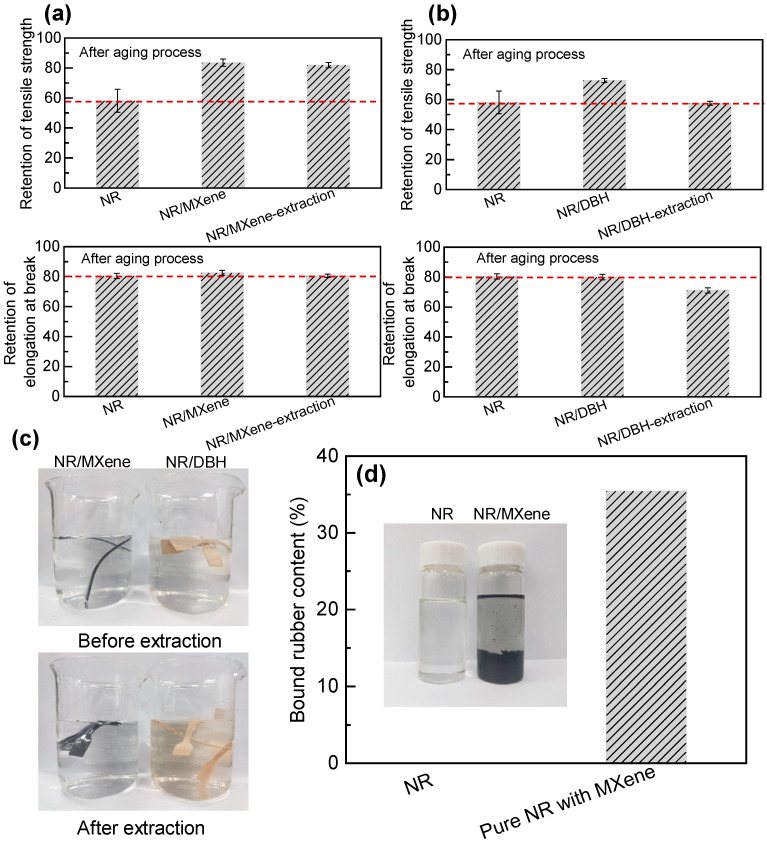
Superior extraction resistance of MXene in the NR matrix. (**a**) Analysis of tensile strength upon aging process, which contains NR, NR/MXene composite, and NR/MXene composite after hot water extraction. (**b**) Analysis of elongation at break upon aging process, which contains NR, NR/MXene composite, and NR/MXene composite after hot water extraction. (**c**) Images of NR/MXene and NR/DBH before and after hot water extraction. The water upon extraction becomes yellow in NR/DBH, while that shows no obvious change in NR/DBH. (**d**) Bound rubber content of NR and pure NR with MXene.

Long-term stabilization of MXene in the matrix is an important factor for the application. After NR/MXene sample was extracted by 50 °C hot water for three days, we characterized the retention of tensile strength and the retention of elongation at break (Figure 4a). Such retention of properties after hot water extraction are the same as those without hot water extraction. Our group also introduced the conventional antioxidant 1,4-dibenzyloxybenzene (DBH) into the NR matrix (NR/DBH) and uses hot water to extract NR/DBH for three days. For NR/DBH, the retention of tensile strength and retention of elongation at break upon hot water extraction are both lower than that without hot water extraction (Figure 4b). Moreover, such retention of NR/DBH upon extraction is similar to that of NR, which indicates that hot water extraction gradually weakens the anti-aging effect of DBH in the NR matrix. The reason is that it is difficult to extract MXene from the matrix but it is easy to extract DBH from the matrix. For example, the water upon extraction becomes yellow in NR/DBH, while that shows no obvious change in NR/DBH (Figure 4c). To investigate the interaction between MXene and molecular chains, we characterized the bound rubber content of pure NR with MXene (Figure 4d). After samples dissolve in toluene for three days, we barely found bound rubbers in NR, while we observed lots of bound rubbers in NR/MXene. MXene absorbing polymer chains results in the increase of bound rubber content, which is similar to the effects of graphene and carbon black [33,34,35,36,37]. Such interaction between NR and MXene inhibits the migration of MXene toward the surface of the sample, improving the long-term thermo-oxidative effect.

### 3.5. Superiority of MXene in Thermo-Oxidative Resistance 

**Figure 5:**
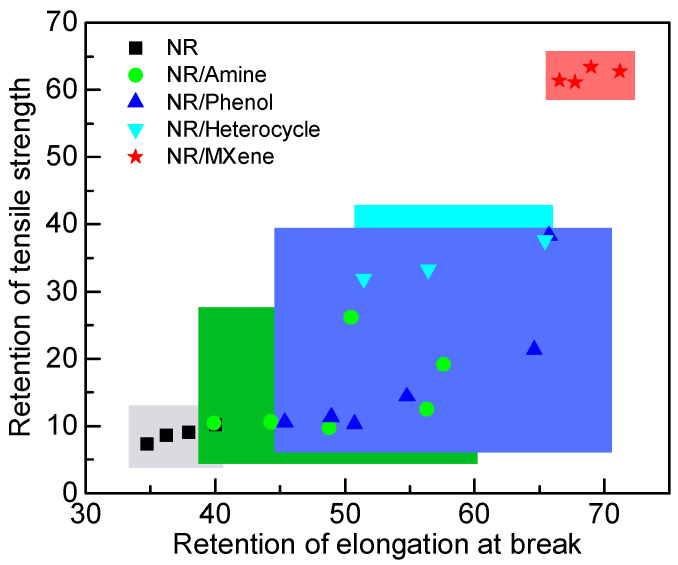
The diagram for retention of tensile strength versus retention of elongation at break upon the long-term thermo-oxidative process. After three days of a thermo-oxidative process, we obtained tensile strength and elongation at break of NR, NR/Amine, NR/Phenol, NR/Heterocycle, and NR/MXene. Amine antioxidants contain N-phenyl-1-naphthylamine, N-cyclohexyl-N’-phenyl-p-phenylenediamine, N-isopropyl-N’-phenyl-4-phenylenediamin, poly(1,2-dihydro-2,2,4-trimethyl-quinoline), N-phenyl-2-naphthylamine, and diphenyl-p-phenylenediamine. Phenol antioxidants contain 1,4-dibenzyloxybenzene, 4,4’-dihydroxybiphenyl, butylated hydroxytoluene, styrenated phenol, polymeric sterically hindered phenol, and 2,2’-methylenebis(6-tert-butyl-4-methyl-phenol). Heterocycle antioxidants contain 2-mercaptobenzimidazole, 2-mercaptobenzimidazole zinc, and methyl-2-mercaptobenzimidazole. Further, we calculated the retention of tensile strength and retention of elongation at break. their interpretation, as well as the experimental conclusions that can be drawn.

Figure 5 depicts the retention of tensile strength as a function of the retention of elongation at break. Our group prepared NR, NR with amine antioxidants (NR/Amine), NR with phenol antioxidants (NR/Phenol), NR with heterocycle antioxidants (NR/Heterocycle), and NR/MXene. After the long-term (three days) thermo-oxidative process, we calculated the retention of tensile strength and retention of elongation at break for all samples (Table S1) and then compared the results in Figure 5. Figure 5 highlights NR/MXene with high retention of tensile strength (63%, 19 MPa) and high retention of elongation at break (70%, 454%) after three days of aging. These results indicate that MXene exhibits superiority in long-term outstanding thermo-oxidative resistance.

## 4. Conclusions

In this work, MXene was proven to play a critical role in achieving elastomer with long-term thermo-oxidative resistance. To investigate the relationship between MXene and thermo-oxidative resistance, our group explored the effect of MXene on oxygen and free radicals in the matrix. The results show that MXene is efficient in absorbing oxygen and the generated free radicals in the NR matrix and can inhibit the diffusion of oxygen toward the interior. Such characteristics of MXene endow NR/MXene with outstanding thermo-oxidative resistance. Moreover, MXene, like graphene and carbon black, absorbs molecular chains, inhibiting the migration of MXene toward the surface of the sample. Therefore, MXene shows long-term outstanding thermo-oxidative resistance in the NR matrix, which far exceeds conventional antioxidants, such as amine antioxidants, phenol antioxidants, and heterocycle antioxidants. This work provides a good guide for the design of elastomer with the long-term outstanding thermo-oxidative resistance.

In further research, we want to regulate the interactions between MXene and elastomers through the modification of interfaces. The aim of our work is to enable elastomers with multifunction properties and high strength.

## Data Availability

The data presented in this study are contained within the article.

## Supplementary Materials

The following are available online at https://www.mdpi.com/2073-4360/13/4/493/s1, Figure S1: XPS patterns of MXenes before (a) and after oxidation (b), Figure S2: XPS patterns of NR and NR/MXene composite. (a) XPS patterns for the interior of NR and NR/MXene composite. (b) XPS patterns for NR and NR/MXene composite upon aging, Figure S3: O/C ratio of NR and NR/MXene composite upon thermo-oxidative process. Figure S4: (a) FTIR spectrum of NR. (b) FTIR spectrum in the range of 3700 to 3100 cm^−1^ of NR. (c) FTIR spectrum in the range of 1750 to 1600 cm^−1^ of NR. (d) FTIR spectrum of NR/MXene composite. (e) FTIR spectrum in the range of 3700 to 3100 cm^−1^ of NR/MXene composite. (f) FTIR spectrum in the range of 1750 to 1600 cm^−1^ of NR/MXene composite. Table S1. The retention of tensile strength and retention of elongation at break for all samples.
