# Supplementary material for: MXene Enabling the Long-Term Superior Thermo-Oxidative Resistance for Elastomers
Source: Polymers (Basel). 2021 Feb 4;13(4):493. doi: 10.3390/polym13040493 (PMC7914804; doi:10.3390/polym13040493)
Supplement: Supplementary file 1 [file polymers-13-00493-s001.pdf]

polymers

Supplementary Material: MXene Enabling the Long-term Superior Thermo-oxidative Resistance for Elastomers

Gui-Xiang Liu , Ya-Dong Yang , Ding Zhu , Yan-Chan Wei , Shuangquan Liao * and Mingchao Luo *

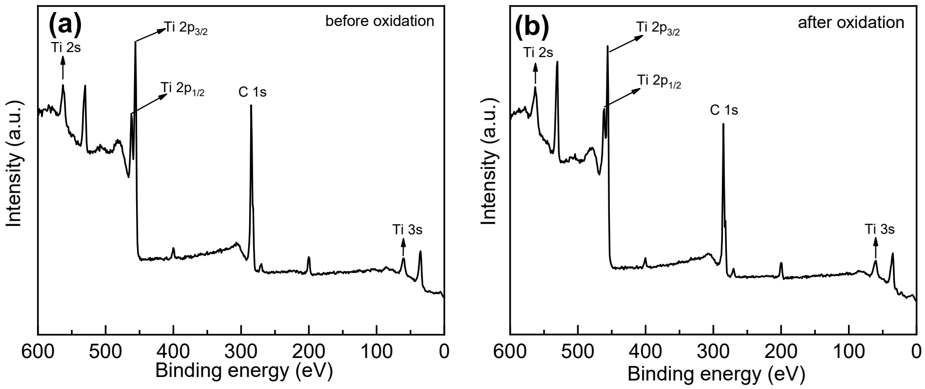

Figure S1. X-ray photoelectron spectroscopy (XPS) patterns of MXenes before (**a**) and after oxidation (**b**).

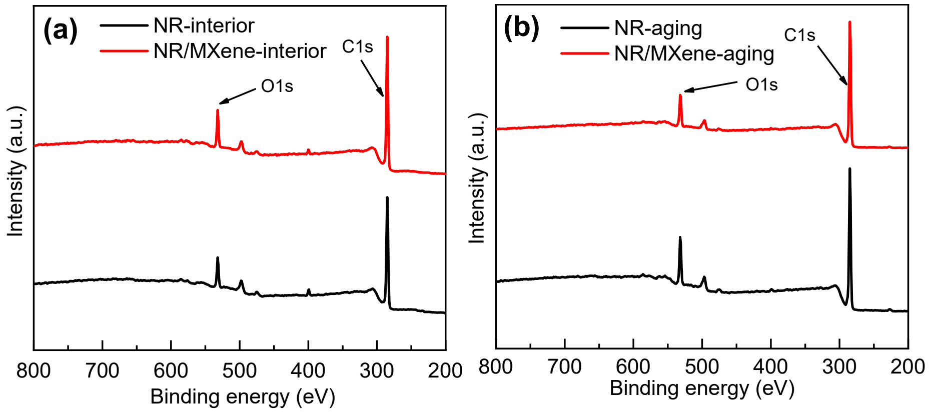

Figure S2. XPS patterns of nature rubber (NR) and NR/MXene composite. (**a**) XPS patterns for the interior of NR and NR/MXene composite. (**b**) XPS patterns for NR and NR/MXene composite upon aging.

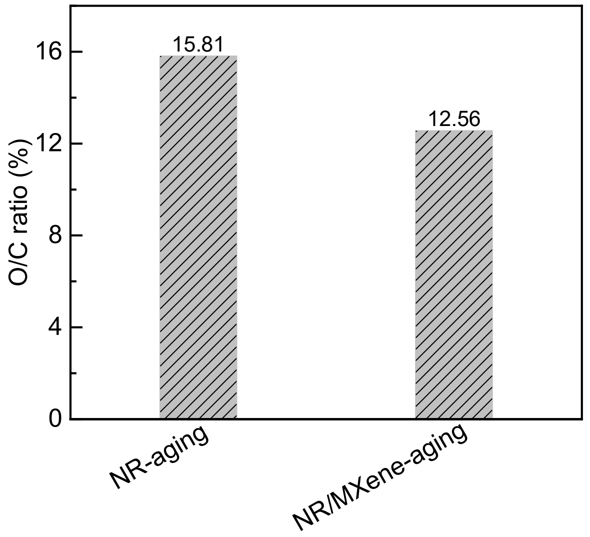

Figure S3. O/C ratio of NR and NR/MXene composite upon thermo-oxidative process.

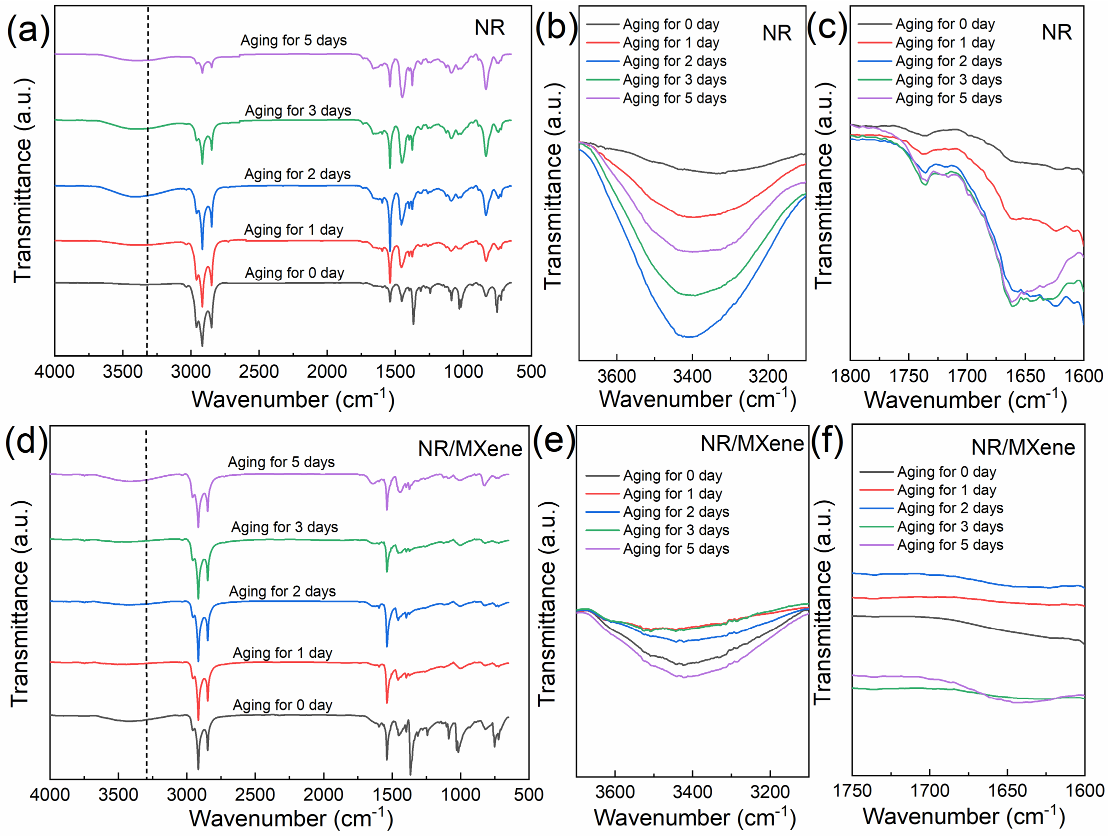

Figure S4 **(a)** Fourier transform infrared (FTIR) spectrum of NR. **(b)** FTIR spectrum in the range of 3700 to 3100 cm^{-1} of NR. **(c)** FTIR spectrum in the range of 1750 to 1600 cm^{-1} of NR. **(d)** FTIR spectrum of NR/MXene composite. **(e)** FTIR spectrum in the range of 3700 to 3100 cm^{-1} of NR/MXene composite. **(f)** FTIR spectrum in the range of 1750 to 1600 cm^{-1} of NR/MXene composite.

Table S1. The retention of tensile strength and retention of elongation at break for all samples.

Sample	Before Aging		After 3 Days Aging		Retention	
	Tensile Strength (Mpa)	Elongation at Break (%)	Tensile Strength (Mpa)	Elongation at Break (%)	Tensile Strength Retention (%)	Elongation at Break Retention (%)
NR-1	27.4	652.4	2.5	247.3	9.1	37.9
NR-2	28.1	683.5	2.1	237.2	7.4	34.7
NR-3	28.1	657.4	2.9	262.9	10.3	40.0
NR-4	28.9	652.2	2.5	237.4	8.7	36.2
N-phenyl-1-naphthylamine	27.1	647.9	3.4	364.8	12.5	56.3
N-cyclohexyl-N'-phenyl-p-phenylenediamine	29.2	694.7	5.6	400.1	19.2	57.6
N-isopropyl-N'-phenyl-4-phenylenediamin	30.4	706.2	7.9	356.2	26.2	50.4
poly(1,2-dihydro-2,2,4-trimethyl-quinoline)	28.4	666.1	2.8	325.1	9.8	48.8
N-phenyl-2-naphthylamine	25.4	674.7	2.7	298.9	10.7	44.3
diphenyl-p-phenylenediamine	24.4	649.0	2.6	258.9	10.5	39.9
1,4-dibenzyloxybenzene	24.8	652.4	9.5	428.6	38.3	65.7
4,4'-dihydroxybiphenyl	27.2	681.1	5.8	439.9	21.3	64.6
butylated hydroxytoluene	26.4	666.4	2.8	302.5	10.6	45.4
styrenated phenol	26.2	673.7	2.7	341.6	10.3	50.7
polymeric sterically hindered phenol	25.4	656.5	2.9	321.7	11.4	49.0
2,2'-methylenebis(6-tert-butyl-4-methyl-phenol)	25.6	648.1	3.7	355.2	14.4	54.8
2-mercaptobenzimidazole	30.1	793.6	11.3	519.0	37.6	65.4
2-mercaptobenzimidazole zinc	28.6	760.1	9.1	391.5	31.9	51.5
methyl-2-mercaptobenzimidazole	29.1	757.3	9.7	427.1	33.3	56.4
NR/MXene-1	31.2	649.8	19.1	439.9	61.2	67.7
NR/MXene-2	32.1	668.1	20.4	461.0	63.4	69.0
NR.MXene-3	31.2	677.6	19.6	482.5	62.8	71.2
NR/MXene-4	30.1	664.7	18.5	442.0	61.4	66.5
